# Successful treatment of metastatic vulvar malignant melanoma with toripalimab: A rare case report and review of the literature

**DOI:** 10.1097/MD.0000000000030239

**Published:** 2022-09-09

**Authors:** Yi-Hui Li, Yang Zhou, Guang-Ju Zhang, Yu-Wei Wang, Jian-Gong Wang, Xiao-Hong Wang, Yu-Feng Li

**Affiliations:** a Department of Chemoradiotherapy, Tangshan People’s Hospital, Tangshan, Hebei Province, China; b The Cancer Institute, Tangshan People’s Hospital, Tangshan, Hebei Province, China.

**Keywords:** combination chemotherapy, immunotherapy, metastatic vulvar malignant melanoma, toripalimab

## Abstract

Vulvar melanoma is a rare and aggressive tumor with a high risk of local recurrence and distant metastasis. The prognosis is poor with a 5-year overall survival rate of only 46.6%. Management of vulvar melanoma remains a clinical challenge. Recent evidences have shown that immune checkpoint inhibitors are effective in the treatment of vulvar melanoma.

**Patient concerns and diagnoses:**

A 63-year-old woman with vulvar malignant melanoma suffered inguinal lymph node metastasis after vulvectomy and chemotherapy. She underwent inguinal lymph node dissection and inguinal radiotherapy. The tumor progressed again and she received immunotherapy.

**Interventions:**

The tumor progressed again, and she was admitted to our hospital and received toripalimab combined with apatinib and abraxane.

**Outcomes:**

After 6 cycles of immunotherapy, the efficacy achieved partial remission. And with toripalimab as maintenance therapy, the patient achieved durable antitumor efficacy and good safety.

**Lessons:**

In this rare case, the patient with metastatic vulvar malignant melanoma had durable antitumor efficacy and good safety when receiving toripalimab.

## 1. Introduction

Malignant melanoma, mainly derived from the basal layer of melanocytes,^[1]^ is a rare and life-threatening malignant tumor. It accounts for approximately 1% of all tumors which can occur in many parts of the body, including the eyes, skin, mouth, anal canal, esophagus, and vulva.^[2]^ Vulvar melanoma is a unique subclass of mucosal melanoma with a distinct biology and mutational profile.^[3]^ Vulvar melanoma differs from common cutaneous melanoma, as ultraviolet light skin exposure is not a causative factor.^[4]^ Meanwhile, it harbors a high rate of c-*KIT* mutations, which distinguishes it from cutaneous melanomas as well as vaginal melanomas.^[5]^ Vulvar melanoma may present as macules, papules, asymmetric borders, or nodules of irregular coloration, and diameter >7 mm.^[4]^ Late-stage features include pigmented, painful, and bleeding lesion, which can be ulcerated at times.^[6]^ Overall, patients with vulvar melanoma have a poor prognosis. However, there is still a lack of standardized treatment guidelines for vulvar melanoma. Metastatic vulvar melanoma patients were typically offered chemotherapy without improving survival.^[5]^ Tyrosine kinase inhibitors may be considered in those patients harboring a *c-KIT* mutation.^[7]^ Immune checkpoint inhibitors have dramatically changed the landscape for treatments in patients with lung cancer, melanoma, renal-cell cancer, and ovarian cancer by producing both durable tumor regression and prolonged disease stabilization.^[8,9]^ Toripalimab is the first domestic programmed cell death protein-1 (PD-1) antibody in China and has received approvals for the treatment of advanced melanoma.^[10]^ Recent evidences have shown that immune checkpoint inhibitors are effective in vulvar melanoma, which is a promising treatment of vulvar melanoma.

## 2. Case report

**Figure 1.**
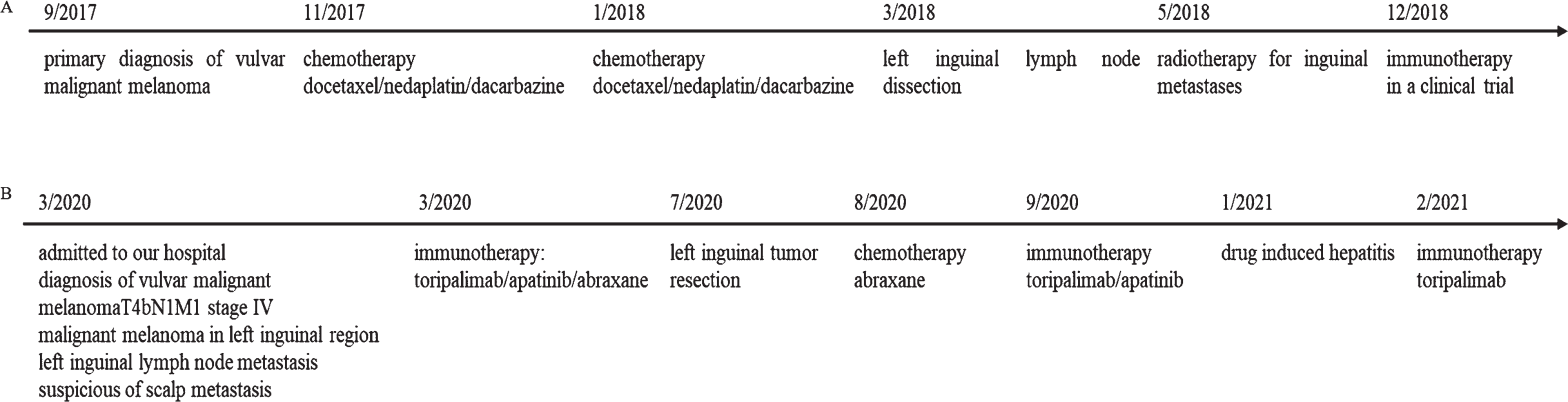
Time axis of the patient’s treatment. (A) Time axis of the treatment in other hospitals. (B) Time axis of the treatment in our hospital.

In September 2017, a 61-year-old woman was admitted to her local hospital for a small vulvar nodule and underwent vulvectomy. The surgical specimen’s pathological findings indicated vulvar malignant melanoma with negative peripheral margin and basal margin, invaded submucosal 1 cm but did not invade the muscular layer. Magnetic resonance imaging of the patient’s brain revealed multiple nodules in the scalp of the left frontal-parietal, suspicious of scalp metastasis. In November 2017, the patient received 2 cycles of chemotherapy of gemcitabine, nedaplatin, and dacarbazine. Due to poor efficacy, then in January 2018, she received 2 cycles of chemotherapy of docetaxel, nedaplatin, and dacarbazine. However, the left inguinal lymph nodes were larger than before. Then she underwent left inguinal lymph node dissection and inguinal radiotherapy in her local hospital. In December 2018, the patient discovered the left inguinal mass again and enrolled in a clinical trial of PD-1 inhibitor in a hospital in Beijing, receiving immunotherapy for 1 year (the specific treatment was unknown). The mass remained stable during treatment (Fig. 1A).

**Figure 2.**
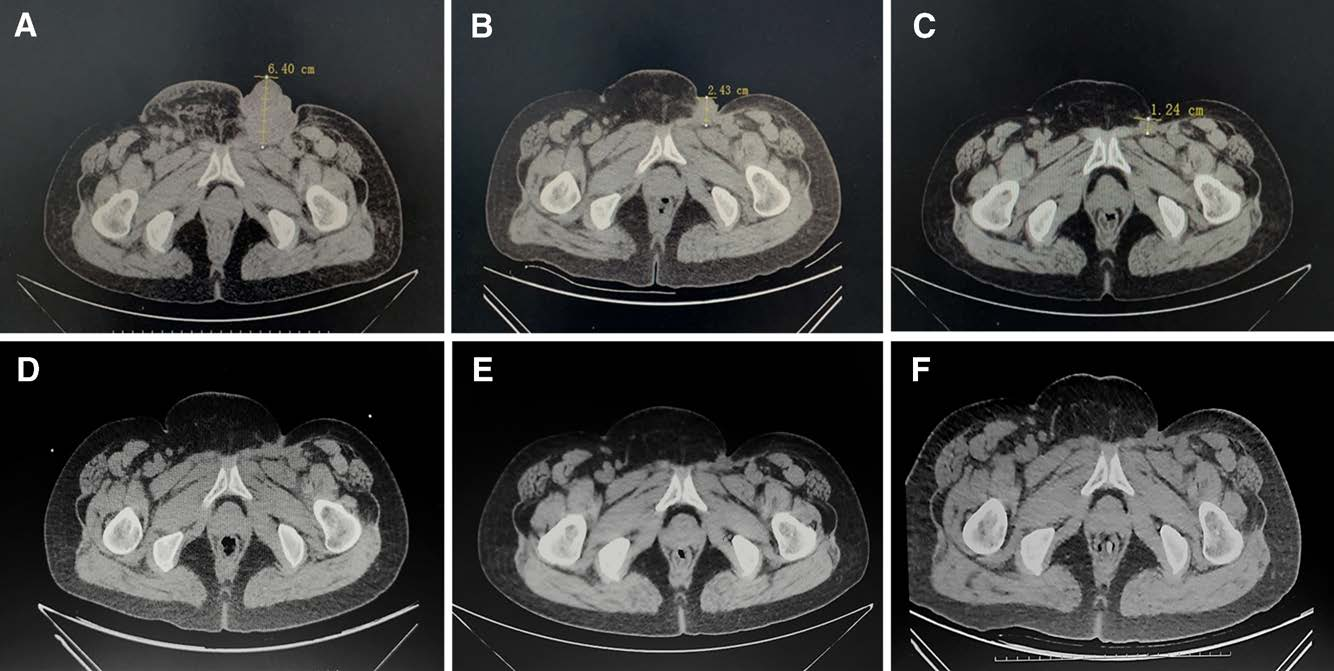
The pelvic CT images of the patient at different stages. (A) A pelvic CT scan at baseline showed a lobulated mass in the left inguinal region. (B) A pelvic CT scan (in May 2020) revealed the mass in the left inguinal region was significantly reduced after 2 cycles of immunotherapy, and the therapeutic effect was evaluated as PR. (C) A pelvic CT scan (in June 2020) demonstrated the mass in the left inguinal region had shrunk further after 4 cycles of immunotherapy, and the efficacy was evaluated as PR. (D) A pelvic CT scan (in December 2020) indicated no mass was in the left inguinal region. (E) A pelvic CT scan (in April 2021) indicated no mass was in the left inguinal region, and the disease was stable. (F) A pelvic CT scan (in August 2021) indicated the disease of the patient remained stable. CT = computed tomography, PR = partial response.

The patient was hospitalized in our hospital in March 2020 for continuous enlargement of the left inguinal mass, accompanied by redness and itching of the vulva for 2 months. Bilateral inguinal ultrasound showed multiple hypoechoic nodules in bilateral inguinal region (considering enlarged lymph nodes). Computed tomography (CT) scan revealed a lobulated mass in the left inguinal region, multiple enlarged lymph nodes in bilateral pelvic wall and inguinal region, and nodules in the left frontal-parietal (Fig. 2A). The patient was diagnosed with vulvar malignant melanoma T4bN1M1 stage IV, malignant melanoma in left inguinal region, left inguinal lymph node metastasis, suspicious of scalp metastasis. In March 2020, she received 6 cycles of immunotherapy: toripalimab 240 mg D1 q3w, apatinib (orally) 250 mg qd, and abraxane 300 mg D2 q3w. The CT scan showed that the mass was significantly reduced and the therapeutic effect was evaluated as partial remission (Fig. 2B and C). In July 2020, left inguinal tumor resection was performed, and the postoperative pathology was consistent with metastatic malignant melanoma. In August 2020, she was treated with abraxane 300 mg and in September she continued to receive 5 cycles of immunotherapy: toripalimab 240 mg and apatinib 250 mg. CT scan suggested no mass in the left inguinal region, and the lymph nodes in the inguinal region were stable (Fig. 2D). In February 2021, the patient presented with jaundice. And liver function tests suggested that the patient’s liver function is abnormal (Table 1). Meanwhile, magnesium isoglycyrrhizinate injection 200 mg (once daily), and polyene phosphatidylcholine 930 mg (once daily) were used for 10 days to protect the liver. The ultrasound-guided puncture biopsy of liver reported mild cholestatic hepatitis and mild chronic drug-induced hepatitis. Considering that hepatitis may be caused by apatinib, apatinib was ceased. From February 2021 to May 2021, she received 4 cycles of toripalimab 240 mg as maintenance therapy. In April, a CT scan revealed no mass in the left inguinal region, and the lymph nodes in the inguinal region remained stable (Fig. 2E). Up to now (Fig. 2F), the patient did not show any evidence of relapse or metastasis after immunotherapy (Fig. 1B).

**Table 1:** The results of liver function tests.

	Value at presentation	Reference
ALT	80 U/L	0–40
AST	261 U/L	0–35
S/L	3.26	0.8–1.5
TBIL	119.2 μmol/L	3.4–20.5
DBIL	99.9 μmol/L	0–8.6
IBIL	19.3 μmol/L	0–15
GGT	802 U/L	7–45
ALP	1250 U/L	35–135
TP	76.6 g/L	65–85
ALB	38.8 g/L	40–55
GLB	37.8 g/L	20–40
A/G	1.03	1.2–2.4
CHE	9235 U/L	4500–13,000

The table demonstrates the liver function tests for the patient.

ALB = albumin, ALP = alkaline phosphatase, ALT = alanine aminotransferase, AST = aspartate transaminase, CHE = cholinesterase, DBIL = direct bilirubin, GGT = γ-glutamyl transpeptidase, GLB = globulin, IBIL = indirect bilirubin, TBIL = total bilirubin, TP = total protein.

## 3. Discussion

Vulvar malignant melanoma, a rare gynecological malignant tumor, accounts for 1% of all melanoma diagnosed in women and 5% of all vulvar malignancies.^[11]^ Vulvar malignant melanoma is categorized as mucosal melanomas, which have a significantly worse prognosis compared with cutaneous melanomas.^[12]^ The 5-year overall survival (OS) rate in vulvar melanoma is only 46.6% compared with 92% in cutaneous melanoma.^[13]^ Surgical excision is the primary treatment for localized melanoma.^[5]^ Woman with advanced or metastatic vulvar melanoma usually received chemotherapy before the era of immune checkpoint inhibitors. With the advent of immune checkpoint inhibitors, the treatment of advanced or metastatic melanoma has changed dramatically. The PD-1 inhibitors pembrolizumab and nivolumab have been approved for the treatment of advanced melanoma. However, a pooled analysis of clinical trials suggested mucosal melanomas had lower efficacy to nivolumab and pembrolizumab compared with cutaneous melanomas.^[14,15]^ In addition, the data on the treatment of vulvar melanoma with immune checkpoint inhibitors are scarce. In a retrospective study including 28 cases of vulvar melanoma and 4 cases of vaginal melanoma, 13 patients (12 cases of vulvar melanoma and 1 case of vaginal melanoma) were treated with immune checkpoint inhibitors; the best overall objective response rate in 13 patients was 30.8%, the median progression-free survival was 4.0 months, and the median OS was 17 months.^[11]^

Toripalimab, a selective recombinant, humanized monoclonal antibody against PD-1, prevents binding of PD-1 with programmed death ligands 1 and 2.^[16]^ In 2018, toripalimab was approved in China for use in the treatment of unresectable or metastatic melanoma that has failed previous systemic therapy.^[16]^ In a nonrandomized, open-label phase 1b trial, 33 chemotherapy naïve advanced mucosal melanoma patients received toripalimab combined with axitinib, 20 patients achieved an objective response, the confirmed objective response rate was 51.5%, and the disease control rate was 84.4%. The median progression-free survival was 9.1 months, and the median OS was not reached by the cutoff date.^[17]^ Unfortunately, only 7 patients with genital tract melanoma were enrolled in this study, and whether there were patients with vulva melanoma was unknown. Furthermore, in clinical trials conducted in China, toripalimab was generally well tolerated. A phase I study of toripalimab in 25 patients demonstrated that the commonest treatment-related adverse events (TRAEs) were fatigue (64.0%) and rash (24.0%). No grade 3 or higher TRAEs were observed.^[18]^ In a study that enrolled 36 patients treated with toripalimab, 100% of patients had TRAEs with most adverse events being grade 1 or 2, and ≥grade 3 TRAEs occurred in 36%.^[19]^ In this rare case, the patient with metastatic vulvar malignant melanoma had durable antitumor efficacy and good safety when receiving toripalimab. However, there are still limitations of this case report because of some undefined treatments in the patient’s history. The patient did not have a sentinel node procedure during first surgery, so whether there was lymph node metastasis was unknown. At the same time, chemotherapy after surgical resection was not standardized enough and the first-line treatment was not effective. Moreover, vulvar malignant melanoma always harbors a high rate of *c-KIT* mutations. However, the patient refused to do molecular analyses, the *c-KIT* mutation was unknown. If the patient received more standardized treatment initially, better outcomes may be achieved.

Vulvar melanoma is a rare and life-threatening malignant tumor of the female genitourinary tract. The management of vulvar melanoma is still a challenge. Recent evidences show that immune checkpoint inhibitors are effective in vulvar melanoma, and this case add evidence of the efficacy and safety of immune checkpoint inhibitors for vulvar melanoma. However, more clinical trials are needed to provide convincing evidence.

### Author contributions

Yi-Hui Li, Yang Zhou, Jian-Gong Wang, Xiao-Hong Wang and Yu-Feng Li participated in the design of the study, and critical revision of the manuscript. Yi-Hui Li, Guang-Ju Zhang and Yu-Wei Wang were responsible for clinical management and collected the data. Yi-Hui Li, Yang Zhou, Jian-Gong Wang performed the analysis. Zhou Yang drafted the manuscript.
